# Combining T1rho and advanced diffusion MRI for noninvasively staging liver fibrosis: an experimental study in rats

**DOI:** 10.1007/s00261-024-04327-3

**Published:** 2024-04-12

**Authors:** Yiwan Guo, Tingting Guo, Chen Huang, Peng Sun, Zhigang Wu, Ziwei Jin, Chuansheng Zheng, Xin Li

**Affiliations:** 1 Department of Radiology, Union Hospital, Tongji Medical College, Huazhong University of Science and Technology, Wuhan, 430022 China; 2 Hubei Province Key Laboratory of Molecular Imaging, Wuhan, 430022 China; 3 Clinical & Technical Support, Philips Healthcare, No. 1628, Zhongshan Road, Wuhan, China

**Keywords:** Liver fibrosis, Multiparametric MRI, T1ρ, DKI, IVIM

## Abstract

**Purpose:**

To investigate the value of imaging parameters derived from T1 relaxation times in the rotating frame (T1ρ or T1rho), diffusion kurtosis imaging (DKI) and intravoxel incoherent motion (IVIM) in assessment of liver fibrosis in rats and propose an optimal diagnostic model based on multiparametric MRI.

**Methods:**

Thirty rats were divided into one control group and four fibrosis experimental groups (*n* = 6 for each group). Liver fibrosis was induced by administering thioacetamide (TAA) for 2, 4, 6, and 8 weeks. T1ρ, mean kurtosis (MK), mean diffusivity (MD), perfusion fraction (*f*), true diffusion coefficient (*D*), and pseudo-diffusion coefficient (D*) were measured and compared among different fibrosis stages. An optimal diagnostic model was established and the diagnostic efficiency was evaluated by receiver operating characteristic (ROC) curve analysis.

**Results:**

The mean AUC values, sensitivity, and specificity of T1ρ and MD derived from DKI across all liver fibrosis stages were comparable but much higher than those of other imaging parameters (0.954, 92.46, 91.85 for T1ρ; 0.949, 92.52, 91.24 for MD). The model combining T1ρ and MD exhibited better diagnostic performance with higher AUC values than any individual method for staging liver fibrosis (≥ F1: 1.000 (0.884–1.000); ≥ F2: 0.935 (0.782–0.992); ≥ F3: 0.982 (0.852–1.000); F4: 0.986 (0.859–1.000)).

**Conclusion:**

Among the evaluated imaging parameters, T1ρ and MD were superior for differentiating varying liver fibrosis stages. The model combining T1ρ and MD was promising to be a credible diagnostic biomarker to detect and accurately stage liver fibrosis.

**Graphical abstract:**

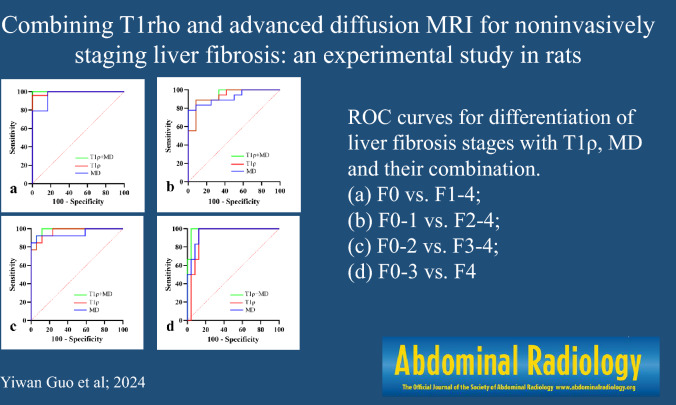

## Introduction

Liver fibrosis is a common feature of various etiologies, such as viral hepatitis, autoimmune hepatitis and biliary disease, and may eventually progress to cirrhosis or hepatocellular carcinoma [1, 2]. Accumulating evidence has suggested that liver fibrosis, especially in the early stage, has the potential to reverse to normal architecture and function [3, 4]. Therefore, it is critical to diagnose and accurately stage liver fibrosis as early as possible.

For decades, liver biopsy has been regarded as the gold standard for the diagnosis and staging of liver fibrosis in clinical settings. However, there are some drawbacks of this invasive method, including painfulness, bleeding, sampling errors and interobserver variability [5]. Therefore, it is not suitable for long-term monitoring or assessing the therapeutic effects of liver fibrosis.

Recently, imaging-based methods, such as ultrasound elastography [6], computed tomography (CT) [7], and magnetic resonance imaging-based techniques [8–17], have shown favorable diagnostic efficiency for staging liver fibrosis with histopathological results as reference standard. Among these advanced methods, MRI is a promising and superior tool with several advantages, including its noninvasive nature, high spatial and soft tissue resolution, as well as multiparameter imaging capability. Over the past two decades, magnetic resonance elastography (MRE) has emerged as a promising approach to stage liver fibrosis [8]. However, considering that MRE is costly, requires dedicated hardware and software, and adds to the patients’ examination time, it is hard to widely used in clinical practice [9]. On the other hand, diffusion-based imaging has the benefit of easy incorporation into routine liver MR imaging.

T1ρ has been reported as a potential approach to detect liver fibrosis since it has high sensitivity to probe slow-motion interactions between motion-restricted water molecules and their local macromolecular environment [12]. In addition, as an extension of the conventional diffusion weighted imaging (DWI), diffusion kurtosis imaging (DKI) is based on the non-Gaussian diffusion model that can account for restricted water diffusion within the complex microstructure of most tissues. It was reported that DKI was feasible for predicting liver fibrosis in rats models [14] and in patients with chronic liver disease [15]. Furthermore, intravoxel incoherent motion (IVIM) imaging, which is a biexponential model for separately assessing the true molecular diffusion and microcirculation perfusion, was also believed to be a useful biomarker in assessment of liver fibrosis [13].

However, conflicting conclusions were obtained when diagnosing liver fibrosis with one of the above mentioned MRI techniques (T1ρ, DKI and IVIM) in some clinical and animal studies [16, 17]. It could be explained by that the different MRI scanners, magnetic field strengths and even various study subjects might result in different conclusions. In addition, due to different mechanisms of T1ρ, DKI, and IVIM in staging liver fibrosis, we would like to figure out whether it could further improve the diagnostic efficiency to combine these imaging models.

Therefore, we designed this experimental study to investigate the efficacy of imaging parameters derived from T1ρ, DKI and IVIM for staging liver fibrosis in rats which were scanned with the same 3.0 T MRI machine. What’s more, we intended to establish an optimal diagnostic model based on multiparametric MRI for staging liver fibrosis.

## Materials and methods

This experimental study was approved by the Ethics Committee of Tongji Medical College, Huazhong University of Science and Technology (Approval number: 20223762). All experimental procedures were in compliance with the ARRIVE guidelines.

### Animal model

Thirty male Sprague–Dawley rats (age 8 weeks, 200 ± 20 g) were included in this experimental study and were randomly divided into four experimental groups and one control group (*n* = 6 for each group). To induce liver fibrosis, the rats in the experimental groups were administered thioacetamide (TAA; Sigma-Aldrich, Spain) dissolved in normal saline by intraperitoneal injection three times a week at a dose of 250 mg/kg for 2, 4, 6, and 8 weeks, respectively [18, 19]. The rats in the control group were administered the normal saline by intraperitoneal injection as the same dose and frequency for 8 weeks. All rats were treated humanely and were provided with enough food and water.

### MRI acquisition

A 3.0 T MRI machine (Ingenia 3.0 T, Philips Healthcare, Best, Netherlands), equipped with an eight-channel phased-array rat coil with a 70-mm diameter (Shanghai Chenguang Medical Technologies, China), was used for scanning. The rats were administered 3% pentobarbital (w/v; 0.2 mL/100 g body weight) anesthesia by intraperitoneal injection before scanning. The rats were placed in the supine position with their heads positioned straight forward. To decrease respiratory motion, their abdomens were secured with a belt.

The following scans were performed on all rats: (1) T1ρ was determined using a turbo field echo (TFE) sequence with spin lock frequency = 350 Hz, spin lock time = 0, 11.67, 23.33, and 35 ms; (2) DKI was performed with single-shot spin-echo-planar sequence using tridirectional motion-probing gradients with 4 b values (0, 800, 1300, and 2000 s/mm^2^); (3) The IVIM sequence was on the basis of the single-shot spin-echo-planar imaging with 11 b values (0, 10, 20, 40, 60, 80, 100, 200, 400, 600, and 800 s/mm^2^). Other detailed scanning parameters were listed in Table 1.

**Table 1:** Scan parameters of T1ρ, DKI, and IVIM

Parameters/Sequences	T1ρ	DKI	IVIM
TR/TE (ms/ms)	7.4/3.6	2363/68	1213/55
FOV (mm^2^)	71 × 71	72 × 72	68 × 68
Flip angle (°)	15	90	90
Matrix	72 × 68	48 × 50	44 × 44
Slice thickness (mm)	4	3	3
Number of slices	4	11	11
Interslice gap (mm)	0.4	0.3	0.3

*TR* repetition time; *TE* echo time; *FOV* field of view

### Image analysis

The acquired images were transferred in DICOM format to the IntelliSpace Portal, version 10 (Philips Healthcare, Netherlands).

The T1ρ maps were generated on a pixel-by-pixel basis from all T1ρ-weighted images with different TSLs according to a monoexponential decay model, which can be described by the equation:

1 $$M_{{{\text{TSL}}}} = M_{0} \cdot{\text{exp}}\left( { - TSL/{\text{T1}}\rho } \right)$$

where *M*_*TSL*_ is magnetization with different spin lock times, *M*_*0*_ denotes magnetization with spin lock time of zero, and *TSL* is the time of the spin lock pulse.

DICOM images from the DKI and IVIM acquisition were postprocessed with a vendor provided ADA (advanced diffusion analysis) tool. For the DKI model, diffusion-weighted signal intensities at multiple b values were fitted with the following equation:

2 $$S\left( b \right) = S_{0} \cdot \exp ( - b{\mkern 1mu} + {\mkern 1mu} D + \, b^{2} \, \cdot D^{2} \cdot K/6)$$

where *S(b)* is the signal intensity at a specific b value, *S*_*0*_ is the signal intensity at b = 0 s/mm^2^, *D* is corrected ADC without Gaussian bias, and *K* is a unitless parameter that represents deviation of water motion from Gaussian diffusion.

The IVIM model and its parameters were fitted according to the following bi-exponential equation:

3 $$S\left( b \right)/S_{0} = f\cdot{\text{exp}}( - {\text{b}}D*) + \, ({1} - f)\cdot{\text{exp}}( - {\text{b}}D)$$

where *S(b)* is the mean signal intensity, *S*_*0*_ is the signal intensity at b = 0 s/mm^2^, and *f* is the perfusion fraction. D* is the perfusion-related diffusion coefficient, and *D* represents the diffusion of the non-perfusing fraction.

All MR images were analyzed by two abdominal radiologists (T.T.G and Y.W.G, with 10 and 5 years of experience in abdominal imaging, respectively) who didn’t know the histopathological results. A total of nine regions of interest (ROIs) measuring 10–12 mm^2^ were manually drawn in the liver parenchyma on the central three continuous sections (three ROIs per section), avoiding artifacts, large vessels, bile ducts, and liver boundaries. The mean values of these parameters, including T1ρ, DKI-associated MD and MK, IVIM-associated *f*, *D*, and D*, were calculated for subsequent analysis.

### Histopathology

The rats of each group at 2, 4, 6, and 8 weeks were humanely sacrificed after each MRI acquisition and the livers were removed. Liver samples were subsequently fixed in phosphate-buffered 10% formalin [20]. Some slices were stained with hematoxylin–eosin (HE) for morphologic analysis of liver parenchyma, while Sirius red staining and α-smooth muscle actin (αSMA) immunohistochemical staining were performed on the other fixed liver tissues to assess the degree of fibrosis [21]. All pathologic specimens were reviewed by a pathologist with more than 10 years of experience in liver pathology. Image analysis software (Image J, version 1.52a; National Institutes of Health) was used to measure the positive areas for Sirius red and αSMA immunohistochemical staining. The fibrosis stages were evaluated by the METAVIR classification system [22], with the following stage definitions: F0 no fibrosis; F1 portal fibrosis without septa; F2 portal fibrosis with a few septa; F3 numerous septa without cirrhosis; and F4 cirrhosis.

### Statistical analysis

All statistical analyses were performed using SPSS 25.0 (Chicago, IL, USA). The Shapiro–Wilk test was used to assess the normality of the data distribution. Quantitative data with a normal distribution were expressed as the mean ± standard deviation and the data with a non-normal distribution were presented as the median and interquartile range. Interobserver reproducibility of imaging parameters was evaluated by the intraclass correlation coefficient (ICC). Imaging parameters with an ICC > 0.75 were included and the data measured by the more experienced reviewer would be used for subsequent analysis. The statistical differences of imaging parameters derived from T1ρ, IVIM and DKI among liver fibrosis stages were determined by one-way analysis of variance (ANOVA) and least significant difference (LSD) test. Spearman rank correlation was used to assess the relationship between imaging parameters and histopathological scores. Receiver operating characteristic (ROC) curve analysis and Delong test were used to evaluate the diagnostic efficiency of different imaging parameters for staging liver fibrosis. *P* < 0.05 was considered statistically significant.

## Results

### Histopathological analysis and liver fibrosis staging

**Fig. 1:**
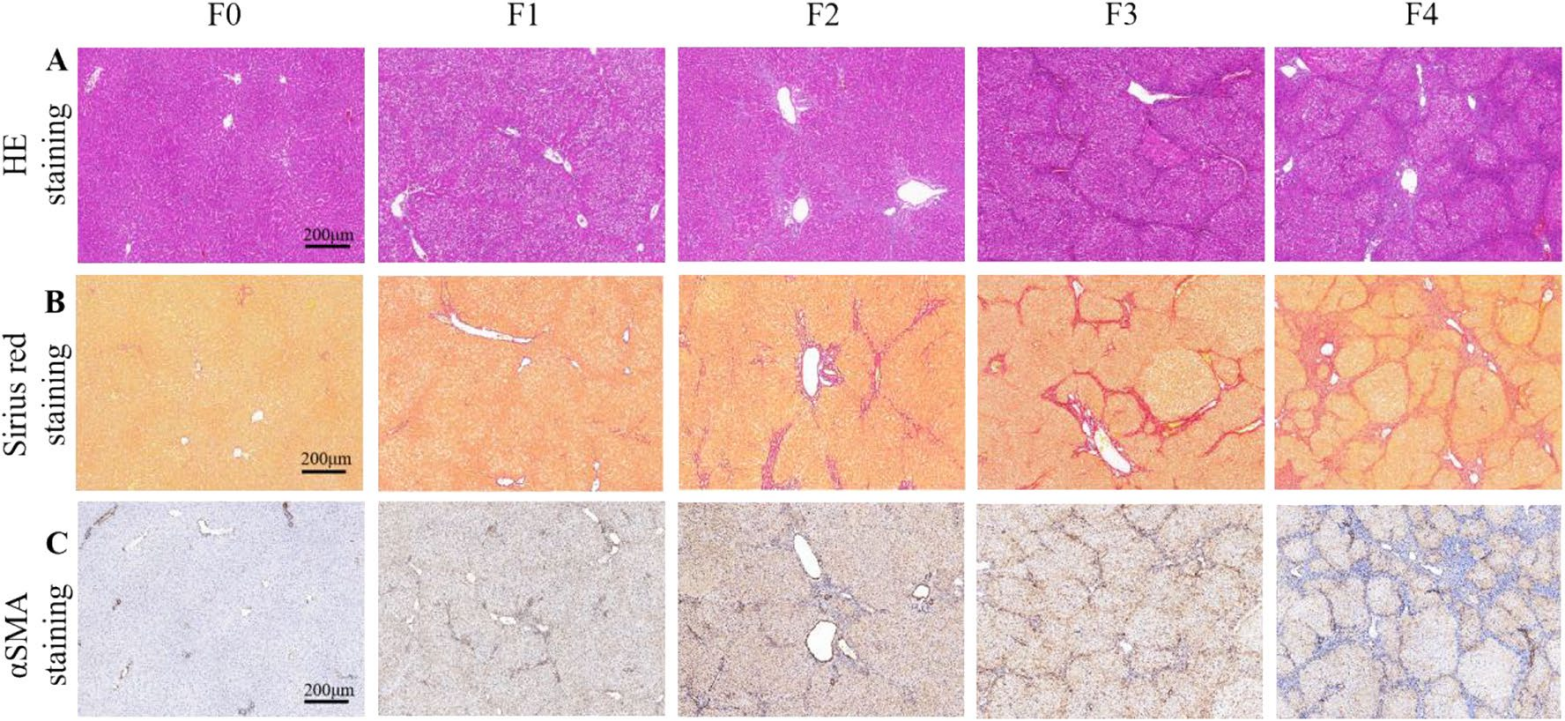
Representative histopathological examples of different liver fibrosis stages. **A**, hematoxylin–eosin (HE) staining; **B**, Sirius red staining; **C**, αSMA staining

HE staining showed that with the progress of liver fibrosis, the arrangement of hepatocyte cords became more and more disordered, and the typical hepatic pseudolobule could be observed in stage F4 fibrosis **(**Fig. 1**)**. In addition, collagen fibers were generated with the formation of liver fibrosis. Sirius red, as a kind of acid dye, can react with collagen fibers to make them red. This phenomenon was further demonstrated by the αSMA immunohistochemical staining. As presented in Table 2, the percentages of positive area for Sirius red and αSMA immunohistochemical staining both increased with the severity of liver fibrosis (*P* < 0.001 for both). According to the histopathological results, there were 6, 6, 5, 7, 6 rats designated into stage F0, F1, F2, F3 and F4, respectively.

**Table 2:** Characteristics of different liver fibrosis stages

Variable	F0 (*n* = 6)	F1 (*n* = 6)	F2 (*n* = 5)	F3 (*n* = 7)	F4 (*n* = 6)	*P* value
T1ρ (ms)	31.47 ± 1.23	34.31 ± 1.31^#^	34.48 ± 0.85^#^	36.99 ± 2.01^#^*^†^	38.59 ± 1.20^#^*^†^	< 0.001
MK	0.91 ± 0.03	0.94 ± 0.04	0.95 ± 0.11	0.98 ± 0.07	1.05 ± 0.08^#^*^†^	0.015
MD (× 10^–3^ mm^2^/s)	1.67 ± 0.14	1.43 ± 0.10^#^	1.39 ± 0.13^#^	1.26 ± 0.11^#^*	1.17 ± 0.05^#^*^†^	< 0.001
*f*	0.27 ± 0.04	0.22 ± 0.05^#^	0.22 ± 0.06^#^	0.19 ± 0.05^#*†^	0.15 ± 0.03^#*†^	< 0.001
*D* (× 10^–3^ mm^2^/s)	0.94 ± 0.09	0.90 ± 0.05	0.83 ± 0.06^#^	0.74 ± 0.07^#^*^†^	0.74 ± 0.08^#^*^†^	< 0.001
D* (× 10^–3^ mm^2^/s)	90.18 ± 6.42	92.47 ± 4.40	89.76 ± 6.85	92.70 ± 2.78	93.62 ± 2.05	0.586
Sirius red-positive ratio (%)	2.02 ± 0.04	5.29 ± 1.13^#^	6.57 ± 1.13^#^*	14.50 ± 0.32^#^*^†^	19.77 ± 1.27^#^*^†‡^	< 0.001
αSMA-positive ratio (%)	1.52 ± 0.28	3.97 ± 0.34^#^	6.09 ± 1.11^#^*	11.72 ± 1.56^#^*^†^	16.33 ± 1.10^#^*^†‡^	< 0.001

*T1ρ* T1 relaxation time in the rotating frame; *MK* mean kurtosis; *MD* mean apparant diffusion; *αSMA* α-smooth muscle actin

^#^*P* < 0.05 versus F0

*P < 0.05 versus F1

^†^*P* < 0.05 versus F2

^‡^*P* < 0.05 versus F3

### Interobserver reproducibility of imaging parameters

T1ρ, MK, MD, *f*, *D* and D* exhibited excellent interobserver reproducibility, with ICC values > 0.75 (*P* < 0.001 for all) **(**Table 3**)**. Notably, T1ρ showed the largest ICC value of 0.951 (95% CI 0.900, 0.976; *P* < 0.001).

**Table 3:** Interobserver reproducibility of imaging parameters

Parameters	ICC	95% CI	*P* value
T1ρ	0.951	0.900–0.976	< 0.001
MK	0.843	0.696–0.922	< 0.001
MD	0.918	0.836–0.960	< 0.001
*f*	0.814	0.580–0.915	< 0.001
*D*	0.911	0.822–0.956	< 0.001
D*	0.753	0.541–0.874	< 0.001

*ICC* intraclass correlation coefficient; *CI* confidence interval; *T1ρ* T1 relaxation time in the rotating frame; *MK* mean kurtosis; *MD* mean apparant diffusion

### Changes in imaging parameters

**Fig. 2:**
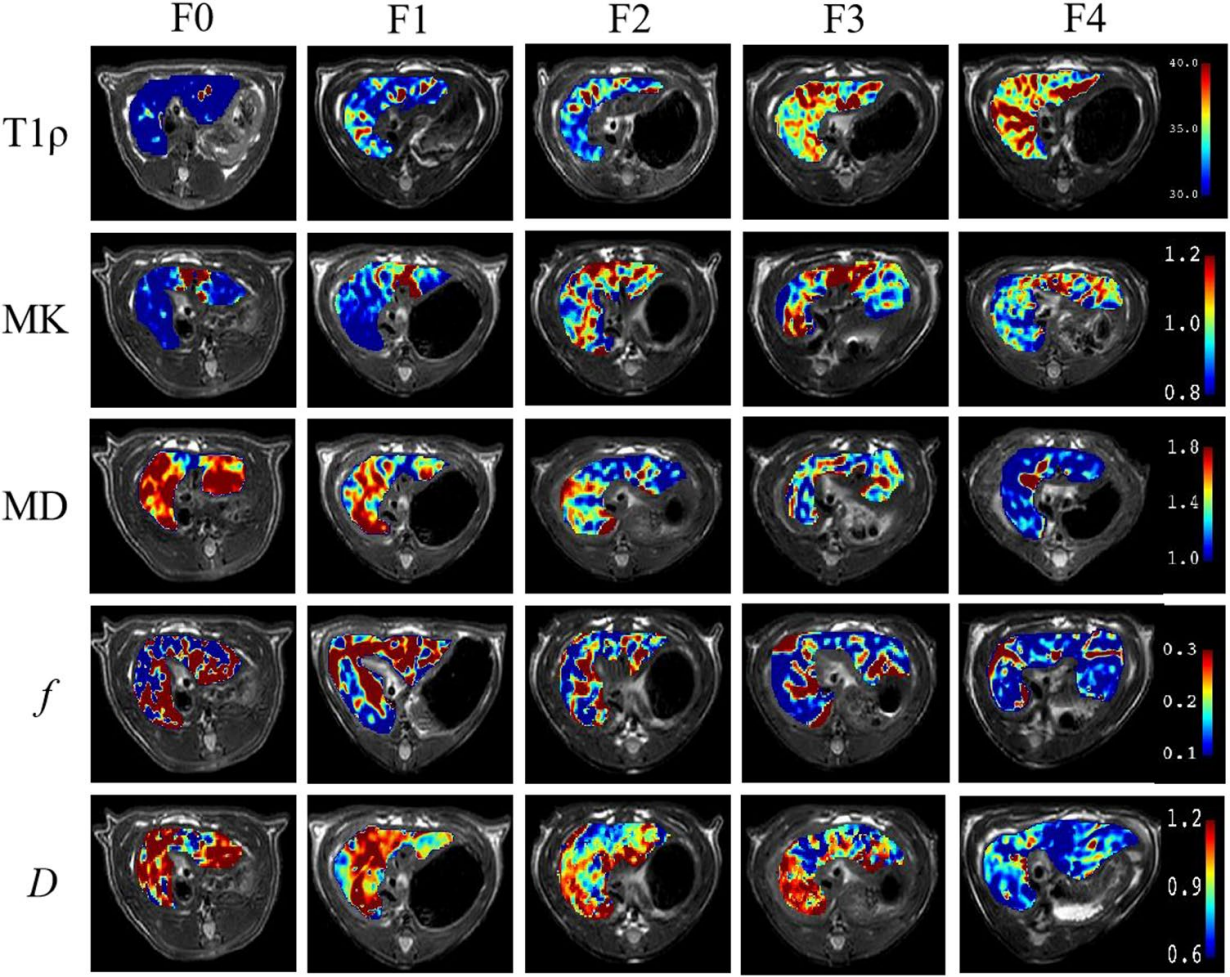
Typical maps of imaging parameters derived from T1ρ, DKI, and IVIM with different fibrosis stages

All imaging parameters were in accordance with normal distribution. According to the ANOVA test, there was no statistical difference of D* among all liver fibrosis stages (*P* = 0.586). Thus, D* was excluded from subsequent analysis. Typical maps of other imaging parameters derived from T1ρ, DKI and IVIM with different fibrosis stages were shown in Fig. 2.

As presented in Table 2, T1ρ and MK values increased with the progression of liver fibrosis (*P* < 0.05 for both). Mean T1ρ values for stage F0-F4 were 31.47 ± 1.23 ms, 34.31 ± 1.31 ms, 34.48 ± 0.85 ms, 36.99 ± 2.01 ms, and 38.59 ± 1.20 ms, respectively. For different fibrosis stages of T1ρ values, the ANOVA with LSD post-hoc test showed that F0 versus F1-4, F1 versus F3-4, and F2 versus F3-4 differed significantly from one another (*P* < 0.05). Mean MK values for stage F0-4 were 0.91 ± 0.03, 0.94 ± 0.04, 0.95 ± 0.11, 0.98 ± 0.07, 1.05 ± 0.08, respectively. Among all stage comparison pairs analyzed by ANOVA with the LSD post-hoc test, F0 versus F4, F1 versus F4, and F2 versus F4 differed significantly from one another.

Conversely, MD, *f* and *D* values generally decreased with the progression of liver fibrosis, especially for MD (*P* < 0.001 for all) (Table 2). And the mean MD values for F0-4 were (1.67 ± 0.14) × 10^–3^ mm^2^/s, (1.43 ± 0.10) × 10^–3^ mm^2^/s, (1.39 ± 0.13) × 10^–3^ mm^2^/s, (1.26 ± 0.11) × 10^–3^ mm^2^/s, and (1.17 ± 0.05) × 10^–3^ mm^2^/s. According to the ANOVA with LSD post-hoc test, F0 versus F1-4, F1 versus F3-4, and F2 versus F4 all had significant difference for MD (*P* < 0.05).

### Correlations between imaging parameters and histopathological scores

As presented in Table 4, there were strong correlations between T1ρ, MD, and *D* and the histopathological scores (Sirius red and αSMA-positive ratios). Spearman correlation coefficients of T1ρ, MD, and *D* versus Sirius red-positive ratios were 0.899, − 0.858, and − 0.787, respectively (*P* < 0.001 for all). Correlation coefficients of T1ρ, MD, and *D* versus αSMA-positive ratios were 0.869, − 0.828, and − 0.758, respectively (*P* < 0.001 for all). MK and *f* showed moderate correlations with histopathogical scores. Spearman correlation coefficients of MK and *f* versus Sirius red-positive ratios were 0.643 and − 0.675, respectively (*P* < 0.001 for both) and versus αSMA-positive ratios were 0.604 and − 0.689, respectively (*P* < 0.001 for both). The results suggested that the correlation coefficients of T1ρ and MD versus histopathological scores were higher and comparable.

**Table 4:** Correlations between imaging parameters and histopathological scores

Parameters	Sirius red-positive ratios		αSMA-positive ratios	
	Coefficient (r)	*P* value	Coefficient (r)	*P* value
T1ρ	0.899	< 0.001	0.869	< 0.001
MK	0.643	< 0.001	0.604	< 0.001
MD	− 0.858	< 0.001	− 0.828	< 0.001
*f*	− 0.675	< 0.001	− 0.689	< 0.001
*D*	− 0.787	< 0.001	− 0.758	< 0.001

*T1ρ* T1 relaxation time in the rotating frame; *MK* mean kurtosis; *MD* mean apparant diffusion; *αSMA* α-smooth muscle actin

### ROC curve analysis

**Fig. 3:**
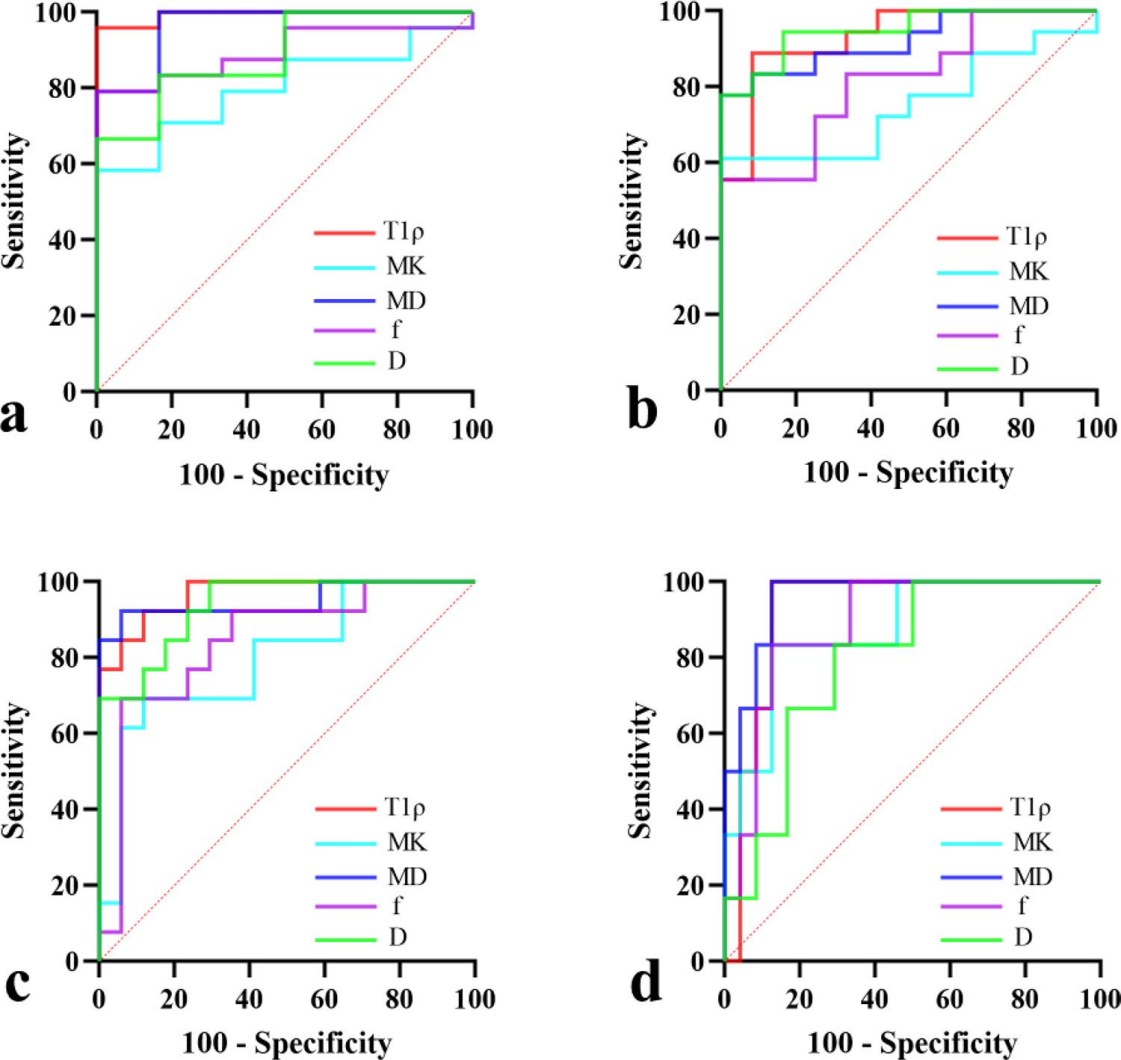
ROC curves for differentiation of liver fibrosis stages with T1ρ, MK, MD, *f*, and *D*. **a** F0 vs. F1-4; **b** F0-1 vs. F2-4; **c** F0-2 vs. F3-4; **d** F0-3 vs. F4

Figure 3 and Table 5 depicted the diagnostic efficiency of T1ρ, MK, MD, *f*, and *D* in differentiating fibrosis stages. The mean AUC values, sensitivity and specificity for various liver fibrosis stages (F0 vs. F1-4, F0-1 vs. F2-4, F0-2 vs. F3-4, F0-3 vs. F4) were summarized in Table 6. According to the ROC curve analysis, T1ρ and MD had relatively better diagnostic efficiency across all fibrosis stages than MK, *f*, and *D*. The mean AUC values for T1ρ and MD were 0.954 and 0.949, while the mean AUC values for MK, *f*, and *D* were 0.806, 0.861, and 0.894, respectively. In addition, the average sensitivity and specificity across all fibrosis stages of T1ρ and MD were also higher than those of MK, *f*, and *D*, which were 92.46 and 91.85 for T1ρ, 92.52 and 91.24 for MD, respectively. However, when comparing AUC values of these imaging parameters across all fibrosis stages, the Delong test suggested that only T1ρ versus MK in differentiating F0 vs. F1-4 and *D* versus MK in differentiating F0-1 vs. F2-4 had statistical significance (*Z* = 2.316, *P* = 0.021; *Z* = 2.425, *P* = 0.015; respectively).

**Table 5:** Diagnostic efficiency of imaging parameters in differentiating liver fibrosis stages

	Parameters	AUC (95% CI)	Sensitivity	Specificity	*P* value
F0 vs. F1-4	T1ρ	0.993 (0.871–1.000)	95.83	100.00	< 0.001
	MK	0.799 (0.613–0.922)	58.33	100.00	< 0.001
	MD	0.965 (0.826–0.999)	100.00	83.33	< 0.001
	*f*	0.896 (0.729–0.977)	79.20	100.00	< 0.001
	*D*	0.889 (0.720–0.974)	83.33	83.33	< 0.001
F0-1 vs. F2-4	T1ρ	0.931 (0.775–0.991)	88.89	91.67	< 0.001
	MK	0.750 (0.559–0.889)	61.11	100.00	0.006
	MD	0.921 (0.763–0.988)	77.78	100.00	< 0.001
	*f*	0.815 (0.631–0.932)	55.56	100.00	< 0.001
	*D*	0.949 (0.802–0.996)	94.44	83.33	< 0.001
F0-2 vs. F3-4	T1ρ	0.968 (0.830–0.999)	92.31	88.24	< 0.001
	MK	0.801 (0.615–0.923)	69.23	88.24	< 0.001
	MD	0.950 (0.803–0.996)	92.31	94.12	< 0.001
	*f*	0.842 (0.663–0.948)	69.23	94.12	< 0.001
	*D*	0.937 (0.784–0.993)	100.00	70.59	< 0.001
F0-3 vs. F4	T1ρ	0.924 (0.766–0.989)	100.00	87.50	< 0.001
	MK	0.875 (0.703–0.967)	83.33	87.50	< 0.001
	MD	0.958 (0.815–0.998)	100.00	87.50	< 0.001
	*f*	0.889 (0.720–0.974)	83.33	87.50	< 0.001
	*D*	0.799 (0.613–0.922)	83.33	70.83	< 0.001

*AUC* area under the ROC curve; *CI* confidence interval; *T1ρ* T1 relaxation time in the rotating frame; MK, mean kurtosis; MD, mean apparant diffusion

**Table 6:** The mean diagnostic values of imaging parameters across all fibrosis stages (F0 vs. F1-4; F0-1 vs. F2-4; F0-2 vs. F3-4; F0-3 vs. F4)

Parameters (mean value for all stages)	AUC	Sensitivity	Specificity
T1ρ	0.954	92.46	91.85
MK	0.806	68.00	93.94
MD	0.949	92.52	91.24
*f*	0.861	71.83	95.41
*D*	0.894	90.28	77.02

*AUC* area under the ROC curve; *T1ρ* T1 relaxation time in the rotating frame; *MK* mean kurtosis; *MD* mean apparant diffusion

**Fig. 4:**
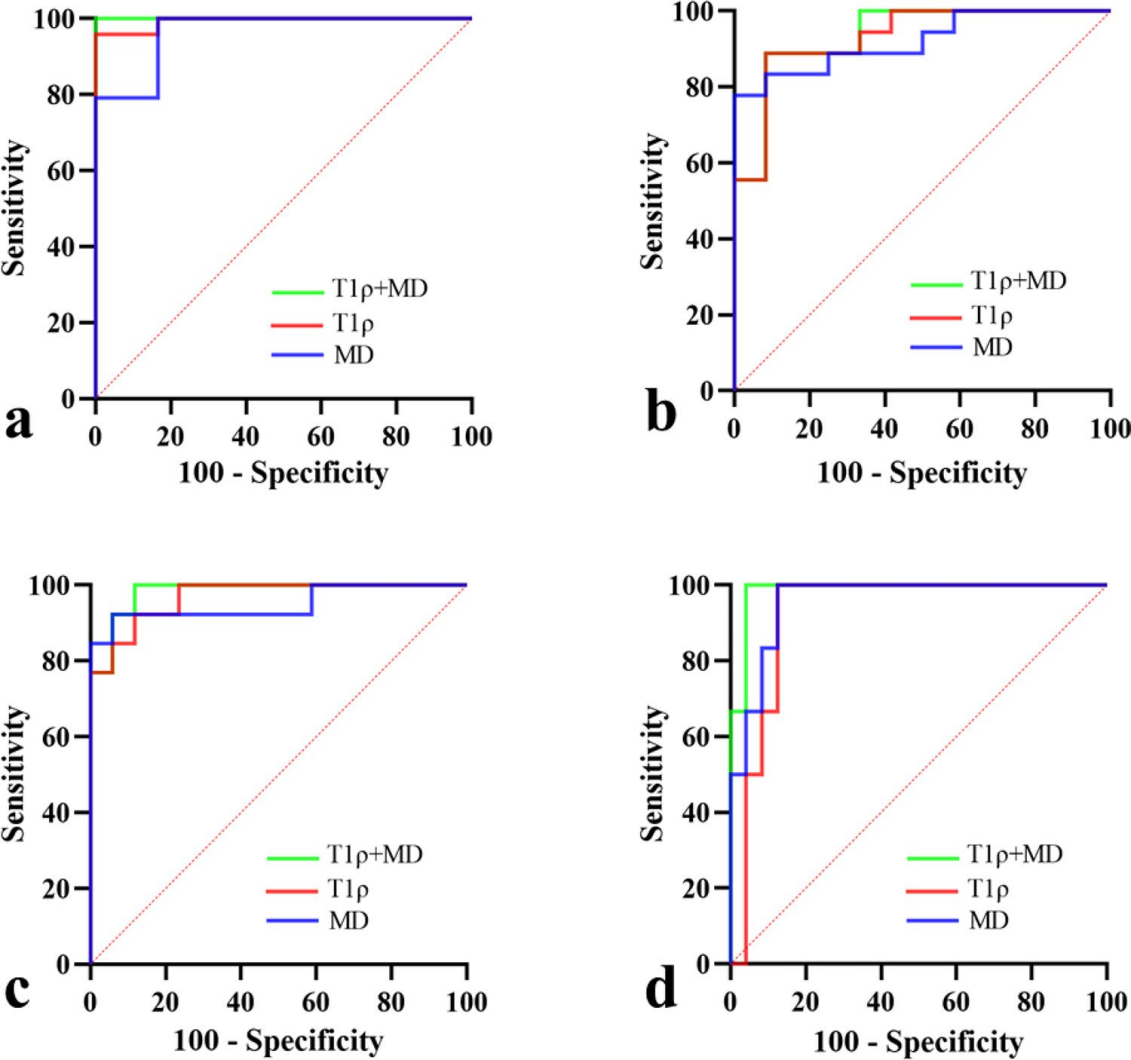
ROC curves for differentiation of liver fibrosis stages with T1ρ, MD and their combination. **a** F0 vs. F1-4; **b** F0-1 vs. F2-4; **c** F0-2 vs. F3-4; **d** F0-3 vs. F4

Further, we established a logistic regression model combining T1ρ and MD and investigated its diagnostic efficiency in discriminating liver fibrosis stages **(**Fig. 4**)**. We found that the combination model exhibited better diagnostic performance with larger AUC values than any individual method (F0 vs. F1-4: 1.000 (0.884–1.000); F0-1 vs. F2-4: 0.935 (0.782–0.992); F0-2 vs. F3-4: 0.982 (0.852–1.000); F0-3 vs. F4: 0.986 (0.859–1.000)).

## Discussion

In this study, we investigated the diagnostic efficiency of imaging parameters derived from T1ρ, DKI, and IVIM for staging liver fibrosis. It suggested that T1ρ and MD derived from DKI both strongly correlated with the histopathological scores (Sirius red and αSMA-positive ratios) and had relatively better diagnostic efficiency across all fibrosis stages than other imaging parameters. In addition, we established a diagnostic model combining T1ρ and MD, which could further improve the diagnostic efficiency for staging liver fibrosis.

It is known that liver fibrosis is the excessive accumulation of extracellular matrix proteins including collagen that occurs in most types of chronic liver diseases [23] and it has been a major public health burden worldwide [24]. In the clinical practice, many patients diagnosed or suspected as liver fibrosis were unwilling to accept liver biopsy to determine the severity of this disease. As a result, imaging-based methods, especially MRI-related techniques, have been investigated to detect and accurately stage liver fibrosis. T1ρ and diffusion-based imaging including DKI and IVIM are easily incorporated into standard clinical liver MRI since these techniques don’t require excess hardware or software or even dedicated contrast agent. In this experimental study, we used TAA to induce liver fibrosis in rats [18] which exhibited the characteristic histopathological features of different liver fibrosis stages. What’s more, we intended to investigate the relationships between imging parameters derived from T1ρ, DKI, and IVIM and histopathological indicators and evaluate the diagnostic efficiency of these parameters for staging liver fibrosis.

In our study, we found that T1ρ increased with the severity of liver fibrosis and had strong positive correlations with Sirius red and αSMA-positive ratios (*r* = 0.899 and 0.869, *P* < 0.001 for both). These results were consistent with previous studies [12, 25]. As for distinguishing different stages of liver fibrosis (F0-F4), T1ρ also displayed excellent performance with AUC values = 0.924–0.993. In addition, we also found that the ICC value of T1ρ was the largest among these evaluated imaging parameters. These results suggested that T1ρ was more stable and reproducible to be measured and was promising to be used in clinical settings to help detect and stage liver fibrosis. In this study, we used a lower spin lock frequency (350 Hz), which was different from the most previous studies (500 Hz). This discrepancy might lead to different sensitivity to macromolecular composition. We used this setup mainly due to two reasons: firstly, a lower spin lock frequency could reduce the specific absorption rate (SAR) [26]. Since this study was carried out on a 3.0 T MR clinical system, the SAR was much larger than previous studies on 1.5 T MR system. We wanted to apply this same scanning protocol in further human studies. The lower spin lock frequency could not only improve the patients’ experience but also shorten the total scanning time; secondly, T1ρ with low spin lock power also gained interests in previous studies [27, 28], although it was prone to artifacts. Therefore, in this study, we employed an adaptive B1 shimming technique to control B1 inhomogeneity [29].

Diffusion-based imaging techniques, such as DKI, also has been studied to stage liver fibrosis in some animal and clinical studies [14, 15]. MK and MD are characteristic parameters derived from DKI. MK reflects the deviation from an ideal Gaussian curve and has been proposed to measure the complexity of the tissue’s microstructure while MD evaluates the water molecule diffusivity inside tissues [30]. In our study, we found that MD decreased with the progression of liver fibrosis while MK increased. Our histopathological results suggested that with the formation of liver fibrosis, the collagen fibers increased and the arrangement of microstructure in the liver became more disordered. These histopathological findings exactly explained the corresponding changes of MD and MK in our study. In terms of differentiating various liver fibrosis stages, we found that MD was superior to MK in any group. This result was similar to that reported by Sheng [20]. It might be accounted for the technical instability of MK given the respiratory motion artifact and insufficient signal-to-noise ratio at high b values [31].

As a biexponential model for separately assessing the true molecular diffusion and microcirculation perfusion [32], IVIM has been used to evaluated liver cirrhosis [33] or fibrosis [34], as well as for characterizing focal liver lesions [35]. In our study, we found that there was no statistical significance for D* among varying fibrosis stages (*P* = 0.586). This result was consistent with that reported by Liang [36]. In contrast to our findings, some studies suggested that D* decreased with the severity of liver fibrosis [13, 37]. The difference might be accounted for that the acquisition of IVIM parameters depended on filed strength and b values might also influence the result. *D* and* f* in our study were found to decrease with increasing liver fibrosis stages and could differentiate various fibrosis stages. In particular, *D* showed favorable diagnostic efficiency in differentiating early fibrosis (F0-1 vs. F2-4 and F0-2 vs. F3-4), whose AUC values were 0.949 and 0.937, respectively (*P* < 0.001 for both). It suggested that *D* might be helpful to noninvasively detect early liver fibrosis.

When comparing the diagnostic efficiency of parameters derived from T1ρ, DKI, and IVIM for staging liver fibrosis, our study suggested that T1ρ and MD had higher mean AUC values, sensitivity and specificity than others across all fibrosis stages. However, no statistical differences were found among these AUC values except that T1ρ versus MK in differentiating F0 vs. F1-4 and *D* versus MK in differentiating F0-1 vs. F2-4. It might be due to the small sample size in this experimental study.

It is known that T1ρ reflects the macromolecular composition and proton exchange in tissues [38]; while MD not only potentially better reflects water diffusivity in tissues at high b values, but also contains specific information on the non-Gaussian diffusion behavior [23]. Therefore, MD derived from DKI may provide T1ρ with added information. Based on this conception, we established a predictive model combining T1ρ and MD to stage liver fibrosis and compared the diagnostic efficiency with T1ρ or MD alone. The results revealed that the combination model performed better with larger AUC values than any individual parameter across all fibrosis stages (AUC = 0.935–1.000). To the best of our knowledge, no previous studies have reported the value of combining T1ρ and DKI-related parameters to stage liver fibrosis. Our study suggested that the combination model might be a credible diagnostic biomarker to detect and accurately stage liver fibrosis. Further studies with a larger sample size in other species were needed to verify this finding.

Our study has several limitations. First, the sample size in this experimental study was small, which might lead to statistical bias. Second, the rat model of liver fibrosis might not reflect the real pathologic changes of human liver. As is known to us, the liver fibrosis and cirrhosis in humans are often caused by chronic liver disease, such as hepatitis B or C. Third, inflammation, steatosis and iron deposition were also recognized as underlying pathologies and might influence the measurement of MRI parameters. However, these confounding variables were not quantitatively evaluated in this study. Further studies were warranted to investigate and correct the impacts of inflammation, iron deposition and steatosis in the process of liver fibrosis.

Our study suggested that among the evaluated imaging parameters, T1ρ and MD derived from DKI were superior for differentiating varying liver fibrosis stages. The model combining T1ρ and MD was promising to be a noninvasive biomarker to detect and accurately stage liver fibrosis in the clinical practice.

## Data Availability

The data used to support the findings of this study are available from the corresponding author upon request.
